# Does Pro^12^Ala Polymorphism Enhance the Physiological Role of PPAR**γ**2?

**DOI:** 10.1155/2013/401274

**Published:** 2013-07-31

**Authors:** A. C. Pereira, R. Oliveira, A. C. Castro, R. Fernandes

**Affiliations:** ^1^Unit of Molecular Mechanisms of Disease (CISA) and Chemical and Biomolecular Sciences, School of Allied Health Sciences, Polytechnic Institute of Porto (ESTSP-IPP), Portugal; ^2^Center of Pharmacology and Chemical Biopathology (U38-FCT), Medical Faculty, University of Porto, Portugal; ^3^Center for Research in Health Technologies and Information Systems (CINTESIS), Medical Faculty, University of Porto, Portugal; ^4^Biomathematics, Biostatistics and Bioinformatics, ESTSP-IPP, Porto, Portugal

## Abstract

Obesity and type 2 diabetes mellitus (T2D) are two major public health problems that have motivated the scientific community to investigate the high contribution of genetic factors to these disorders. The peroxisome proliferator activated by gamma 2 (PPAR*γ*2) plays an important role in the lipid metabolism. Since PPAR*γ*2 is expressed mainly in adipose tissue, a moderate reduction of its activity influences the sensitivity to insulin, diabetes, and other metabolic parameters. The present study aims to contribute to the elucidation of the impact of the Pro^12^Ala polymorphism associated with T2D and obesity through a meta-analysis study of the literature that included approximately 11500 individuals, from which 3870 were obese and 7625 were diabetic. Statistical evidence supports protective effect in T2D of polymorphism Pro^12^Ala of PPAR*γ*2 (OR = 0.702 with 95% CI: 0.622; 0.791, *P* < 0.01). Conversely the same polymorphism Pro^12^Ala of PPAR*γ*2 seems to favor obesity since 1.196 more chance than nonobese was found (OR = 1.196 with 95% CI: 1.009; 1.417, *P* < 0.004). Our results suggest that Pro^12^Ala polymorphism enhances both adipogenic and antidiabetogenic physiological role of PPAR*γ*. Does Pro^12^Ala polymorphism represent an evolutionary step towards the stabilization of the molecular function of PPAR*γ* transcription factor signaling pathway?

## 1. Introduction 

**Figure 1:**
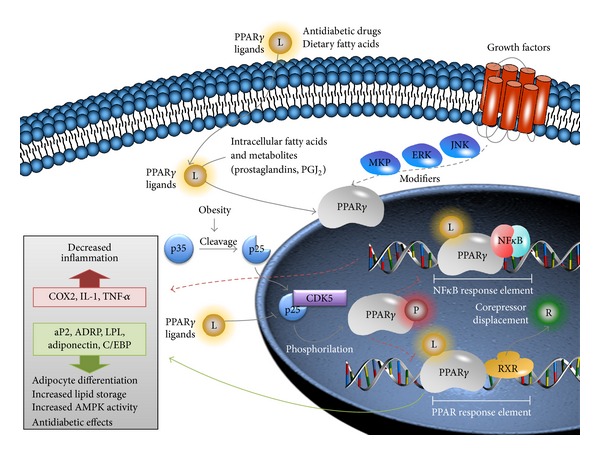
PPAR*γ* transduction pathway. PPAR*γ* has several extracellular and intracellular ligands that include dietary and bioactive lipids. Given its antidiabetogenic role, some PPAR*γ* ligands include antidiabetic drugs such as thiazolidinediones. PPAR*γ* is also modulated by several growth factor transduction pathways such as Jnk/Erk/MKP. It is also known that cyclin-dependent kinase 5 (Cdk5) bond to p25 (a product of the cleavage of p35 in obesity environment) inhibits PPAR*γ* pathway by its phosphorylation. As a transcriptional factor, PPAR*γ* binds to RXR (retinol X receptor) in order to transcribe several genes related to adipocyte differentiation and lipid storage in adipose tissue and increase insulin sensitivity in peripheral tissues by indirect increase of AMP kinase activity as well as several other antidiabetogenic effects. Moreover, PPAR*γ* blocks NF*κ*B signaling, thus reducing proinflammatory cytokines and inflammation.

Peroxisome proliferator-activated receptors (PPARs) are transcription factors from nuclear receptor's protein family that regulate target genes' expression by connecting to response elements of peroxisomes proliferators (PPERs) in regulating sites of each gene. The signal transduction mechanism of these receptors involves retinol X receptor (RXR) and PPARs form heterodimers that regulate the transcription of several genes (Figure 1) [1–3].

Of all PPAR subtypes described, PPAR*α*, PPAR*β*, and PPAR*γ*, the latter is the most studied [4]. The PPAR*γ* gene, extended by a segment of over 150 kb, is localized in chromosome 3, region 3p25, and is constituted by 3 isoforms: PPAR*γ*1, PPAR*γ*2, and PPAR*γ*3, distinguished for generating different promoters and alternative splicing [1, 5, 6].

**Figure 2:**
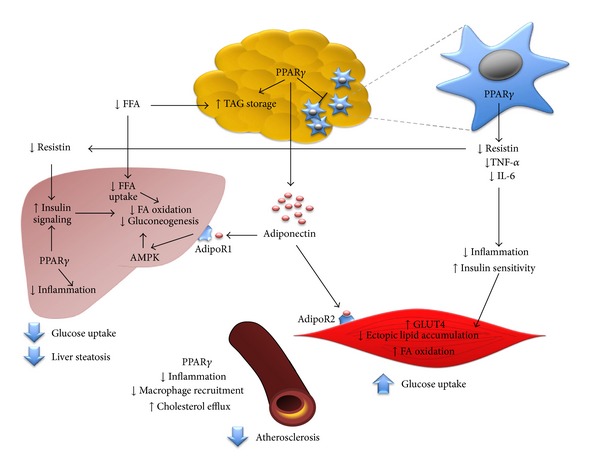
Physiological and metabolic roles of PPAR*γ*. In adipose tissue PPAR*γ* is involved in lipid storage, which reduces circulating free fatty acids (FFA). In adipose tissue PPAR*γ* blocks the proinflammatory role of macrophages as well the secretion of resistin both by macrophages and adipocytes which favors the uptake and metabolism of glucose in the liver, muscle, and other peripherals organs. Adipose tissue also secretes adiponectin, which favors indirectly the activation of AMP kinase in the liver, heart, and muscle. AMPK phosphorylates a number of enzymes involved in fatty acid oxidation and glucose metabolism as well the synthesis and release of GLUT4. In the endothelial tissue, PPAR*γ* favors the cholesterol efflux and at the same time, as consequence of the lowering of inflammatory signals, the recruitment of macrophages, thus delaying the atherosclerosis. Overall, the outcome of downstream effectors results in the decrease of proinflammatory cytokines, free fatty acids, and plasma glucose by increasing its uptake resulting in the increase of generalized insulin sensitivity and reduction of lipid accumulation in the liver, cardiac and skeletal muscle, and arteries as well as the diminishing of chronic inflammation, prothrombotic state, and atherosclerosis.

PPAR*γ*2 is a transcriptional factor responsible for many metabolic and cellular processes, such as cellular growth, differentiation, and metabolism, in response to lipophilic hormones, fatty acids, and its metabolites [1, 2]. It has high affinity to fatty acids specific proliferators and is almost exclusively expressed in the adipose tissue in humans, where it modulates target genes expression involved in adipocytes expression, insulin sensitivity, angiogenesis, and inflammatory processes, among others (Figure 2) [1, 5–7].

To date, several PPAR*γ*2 polymorphisms have been described and the Pro^12^Ala polymorphism has been associated with variations of BMI and insulin sensitivity, depending on the ethnicity, although the reasons for this heterogeneity remain unclear [1, 7–9]. 

Given that PPAR*γ*2 is mainly expressed in adipose tissue and taking in consideration the role of free fatty acids (FFA) and adipokines in insulin regulation, the effect of the Pro^12^Ala polymorphism may be anticipated to measure these factors alterations. Hence, individuals with the Ala12 allele present have been described to have diminished lipoprotein lipase activity [10], which can result in lipoproteins decrease and, consequently, in plasmatic FA decrease, with harm to insulin action in the skeletal muscle [11]. Accordingly, Ala12 allele carriers have been described to possess lower plasmatic FA levels, higher blood flux in adipose tissue and skeletal muscle, and higher insulin sensitivity [12]. Also, insulin suppression resultant from the lipolysis process in adipose tissue is increased in lean individuals as well in T2D patients with the Ala12 allele [13, 14]. However, long term lipolysis inhibition increases adiposity [13]. Still, these may not be the real mechanism or the only mechanism subjacent to the Pro^12^Ala polymorphism effect.

Obesity is classified as a multifactorial chronic disease that consists in body fat accumulation in adipose tissue and is globally recognized as an important public health issue, affecting people of all ages and ethnicity that results in several metabolic complications [15].

Diabetes mellitus is a metabolic disorder of multiple etiologies, characterized by chronic hyperglycemia due to deficient secretion or action of insulin and, like obesity, is considered one of the main heath threats [16].

The present study pretends to contribute to the understanding of the role of the PPAR*γ*2 gene Pro^12^Ala polymorphism in two metabolic conditions, obesity and diabetes mellitus 2 (T2D), through a systematic review and meta-analysis.

## 2. Materials and Methods 

We performed a systematic review and meta-analysis of published works about the presence of particular PPAR*γ* polymorphism such as Pro^12^Ala, as a risk factor for obesity and for T2D, according to Preferred Reporting Items for Systematic Reviews and Meta-Analyses (PRISMA) guidelines.

### 2.1. Selection Criteria and Identification of Studies

The study was conducted according to PRISMA directives. In order to identify the studies, an electronic search, with no linguistic restriction, was conducted, researching two databases, the ScienceDirect and Medline (PubMed). The Boolean search was conducted in both databases according to the following keywords: (1) PPAR*γ* [AND] Pro^12^Ala, (2) Pro^12^Ala [AND] obesity, and (3) Pro^12^Ala [AND] diabetes mellitus 2. 

Two investigators evaluated, independently, the titles and abstracts of the papers identified as of potential relevance in the inclusion criteria. Where a title or abstract could not be included for certainty, a third corrector would help decide whether to reject or not. 

The papers that satisfied the initial inclusion criteria and that were identified as of randomized or quasi-randomized clinical trial conducted in human, and used the Pro^12^Ala polymorphism as a comparison between control groups and obese or diabetic groups, were then used for systematic revision. 

After the papers' analysis, the data of interest was used based on the place where the study was conducted, year of publication, outcome, and number of individuals. 

### 2.2. Statistics

The statistical analysis included false positives, false negatives, true positives, and true negatives to establish sensitivity and specificity and correlate both in a Roc curve, *P* value, and odd ratios (OR), with and with a confidence interval of 95%.

For the data analysis was used the program RevMan version 5.1 and the SPSS Statistics version 17.0.

## 3. Results and Discussion

The research identified 11 studies, from which 6 were related to the presence of Pro^12^Ala polymorphism in obesity and 8 to the presence of Pro^12^Ala polymorphism in T2D.

The studies related to obesity investigated a total of 3870 individuals, with 2260 being obese and 1610 nonobese (Table 1). 

The studies regarding the correlation between Pro^12^Ala polymorphism and T2D investigated a total of 7625, with 4464 being diabetic and 3161 nondiabetic (Table 2).

The major characteristics of the investigated individuals were similar in all included studies, as well as the methodology.

The sensitivity analysis to evaluate population characteristics was not possible due to the small number of included studies. 

Ali and collaborators studied the effect of Pro^12^Ala polymorphism on obesity risk in a Tunisian population, in 2009. They notice a significant difference between male obese individuals and control and pointed out an association between the polymorphism and obesity in nondiabetic male. These individuals also displayed elevated BMI. However this study did not proceed to body fat measurement or its distribution in order to confirm the polymorphism contribution in obesity. Still, it is supported the hypothesis that Pro^12^Ala polymorphism is a relevant marker in nondiabetic Tunisian man obesity, despite a noneffect on the individuals metabolic characteristics [20].

Ereqat and collaborators studied obese Palestinian individuals with T2D. They notice the polymorphism was associated with high plasmatic levels of total cholesterol, with a tendency to increase LDL cholesterol levels. However, there was no significant impact on BMI, triglycerides, or arterial pressure. Hence, they concluded that the Ala12 allele may influence cardiovascular risk, through effect on lipidic metabolism, in obese Palestinian patients with T2D [19]. 

Ghoussaini and collaborators studied the French population with T2D and obesity. Regarding obesity, they demonstrated no association with Pro^12^Ala polymorphism, in children or adults. Still they concluded that the polymorphism confers a reduction in obesity risk [17].

Oh and collaborators conducted their study in the Korean population. They found no significant association between the Pro^12^Ala polymorphism and obesity, hypertension, or dyslipidemia. However, despite the statistical nonsignificance, there is, according to the authors, the possibility of PPAR*γ*2 mutation having a minor effect on obesity, assuming a significant effect if it occurs simultaneously with other genes' mutations or environmental factors [1].

González Sánchez and collaborators were the firsts to conduct a population-based nationwide multicenter study in Spain, suggesting that Pro^12^Ala polymorphism may promote peripheral deposition of adipose tissue. The Ala12 allele frequency was higher on obese male individuals than in lean ones. Men carrying the Ala12 allele had a higher BMI than noncarriers (38.9% versus 21.3%), despite possessing a lower abdominal diameter [18].

Ghoussaini and collaborators studied the French population with obesity and T2D, in 2005. Regarding the T2D study, they affirmed that the polymorphism has a risk role, since a significant association between the polymorphism and T2D was found, with a *P* value of 0.04 and OR = 1.37 (95% CI: 1.02; 1.85) [17]. 

Lindi and collaborators studied a diabetic Finnish population. They were the first to try an association between Pro^12^Ala polymorphism and T2D incidence, in a high-risk population with diminished glucose tolerance, in a longitudinal study. No significant association of the Ala12 allele and T2D incidence in the studied group [21] was found. 

Chistiakov and collaborators analyzed Russian individuals with T2D. Their results suggest that the polymorphism reduces T2D risk, performing a protective role in this pathology. In the studied population, the Pro12 allele contributes to higher risk in developing T2D, according to OR = 1.69 (95% CI: 1.02; 3.03) [22]. 

Oh and collaborators analyzed the association between Pro^12^Ala polymorphism and T2D, in diabetic Koreans. They compared individuals with normal and diminished glucose tolerance and with diabetes. No significant differences were found among Ala12 allele frequency in the study groups, concluding an inexistent significant association between Pro^12^Ala polymorphism and T2D. However, despite these results, they affirm that there is the possibility of an effect, at minor scale, of the polymorphism on the pathology, when associated with other mutations on other genes, as well with environmental factors [1]. 

Bouassida and collaborators studied the association of Pro^12^Ala polymorphism and Tunisian T2D carriers. They concluded that there was no significant differences between diabetic and control groups and that, therefore, the polymorphism does not display a role in the pathology [23]. 

Malecki and collaborators studied the Pro^12^Ala polymorphism with T2D incidence in a polish population. They compared the polymorphism incidence in a diabetic and a nondiabetic (control) group and notice that the Pro12 and Ala12 alleles' frequency were similar (83.5% and 16.5% versus 84.5% and 15.5%, resp., *P* = 0.607), as well as the genotypic distribution. They could not conclude that the polymorphism confers higher risk to T2D susceptibility, as in numerous European studies in Caucasians [24]. 

Pintérová and collaborators, in 2004, wanted to understand the association between the Pro^12^Ala polymorphism and T2D. In order to do so, they studied the Czech population, comparing a study group of 133 diabetic individuals to a nondiabetic control group of 97 individuals. In the study group, 3 individuals (2.26%) were identified as homozygotes for the Ala/Ala genotype, 99 individuals (74.44%) were identified as homozygotes for the Pro/Pro genotype, and 31 individuals (23.31%) were identified as heterozygotic. In the control group, 6 individuals (6.19%) were identified as homozygotes for the Ala/Ala genotype, 61 individuals (62.89%) were identified as homozygotes for the Pro/Pro genotype, and 30 individuals (30.93%) were identified as heterozygotic. The allelic frequency for the Ala allele was lower in the diabetic group (13.91% versus 21.43% for control group, *P* = 0.022). There was no difference (*P* = 0.05) among phenotypic characteristics (BMI, gender) in the studied group regarding genotype Pro^12^Ala. Therefore this study supports the hypothesis that the polymorphism plays an important role in T2D for the Czech population. The results showed that the allelic frequency of for Ala12 is higher in control group than in study group. Overall the polymorphism is associated with reduced risk of T2D, assuming a protective role in the pathology [25]. 

Mori and collaborators analyzed the polymorphism and T2D in a Japanese population. They included in their study 2201 individuals with T2D (study group) and 1212 individuals without the pathology (control group). The allelic frequency of Ala12 was higher in in control group (4.13% versus 2.39% for the study group). They concluded that the polymorphism is associated with reduced risk of T2D development, acting out protectively factor in the pathology [26].

### 3.1. Meta-Analysis Results

The combination of the studies that analyzed the presence of Pro^12^Ala polymorphism in obesity is showed on Table 3. 

Regarding the presence of Pro^12^Ala polymorphism in T2D, the combination of all included studies is presented in Table 4. 

Eleven studies that investigated the Pro^12^Ala polymorphism were included in the present meta-analysis. From these, 6 studies investigated the hypothetical association of the polymorphism and obesity and 8 investigated the hypothetical association of the polymorphism, Tables 1 and 2. The data was then aggregated according to presence/absence of the polymorphism and presence/absence of the pathology. Individuals with the genotype Pro/Pro did not possess the polymorphism and individuals with genotype Pro/Ala (heterozygotes) and genotype Ala/Ala (homozygotes) possess the polymorphism. This way, it was possible to assort all individuals in TN, FN, TP, and FP and, with these data, obtain the sensitivity and specificity graphics (Tables 3 and 4). The analysis of these tables allowed concluding that the studies are not sensitive but very specific.

In Table 3, Ghoussaini and collaborators' study [17] conducted in obese children represented the most sensitive study, with a value of 0.23 (95% CI: 0.19; 0.28). On the other hand, Oh and collaborators [1] had the least sensitive study, with 0.07 (95% CI: 0.03; 0.14). The study with the highest average specificity belongs to Ben Ali and collaborators [20], conducted in 2009, with a specificity value of 0.94 (95% CI: 0.91; 0.97) and the one with the lowest average specificity was conducted by González Sánchez and collaborators, 0.75 (95% CI: 0.68; 0.80).

In Table 4, Lindi and collaborators' study [21] represented the most sensitive study, with a value of 0.34 (95% CI: 0.28; 0.41). On the opposite side, Mori and collaborators [26] had the least sensitive study, with 0.05 (95% CI: 0.04; 0.06). The latter also had the study with the highest average specificity, with 0.92 (95% CI: 0.90; 0.93) and the one with the lowest average specificity was conducted by Chistiakov and collaborators [22], 0.59 (95% CI: 0.55; 0.63).

The results from these tables allowed constructing the respective ROC curve (not shown) and we conclude that the Pro^12^Ala polymorphism is not a good test (regarding sensitivity and specificity) to predict the presence of obesity, since the test line is very close to the random line. The same conclusion can be taken from the analysis of the Pro^12^Ala polymorphism which is not a good test to predict the presence of T2D. 

We also proceed to the determination of the *odds ratio *(OR) and *P* values, by the Chi-squared test. Tables 5 and 6 show the *P* value for obesity and T2D, respectively, and Tables 7 and 8 show the risk estimative (OR) for the variables used in this meta-analysis, with a 95% CI, for obesity and T2D, respectively.

Regarding the association between the polymorphism and obesity, obese individuals possess a 1.196 more chance of carrying the polymorphism than non-obese (OR = 1.196 with 95% CI: 1.009; 1.417. *P* < 0.004).

Concerning the association between the polymorphism and T2D, diabetic individuals possess a 0.702 less chance of carrying the polymorphism than nondiabetic (OR = 0.702 with 95% CI: 0.622; 0.791. *P* < 0.001).

Therefore, the Pro^12^Ala polymorphism from gene PPAR*γ*2 acts out as a risk factor in obesity (*P* < 0.05 and OR = 1.196 with CI values that never include 1) and as a protective factor for T2D (*P* < 0.05 and OR = 0.702 with CI values that never include 1), by the evident statistical significance. 

## 4. Conclusions

Comparing results obtained from this meta-analysis and each of the included studies, we can verify some differences. There are studies that conclude that Pro^12^Ala polymorphism is a risk factor in obesity or T2D, while others conclude the opposite for both pathologies. There are also studies that do not have statistically significant data to support either conclusion.

Ben Ali et al. [20], Ghoussaini et al. [17], Oh et al. [1], and respective collaborators could not associate, from their studies, the polymorphism presence in obesity. Meanwhile González Sánchez and collaborators [18] associated the polymorphism with higher risk of obesity. Ereqat and collaborators [19] associated the polymorphism to lipidic metabolism in obese Palestinian individuals with T2D, concluding that the Ala allele presence may influence cardiovascular risk.

Results regarding the role of the polymorphism in T2D, Boussida et al. [23], Lindi et al. [21], Malecki et al. [24], Oh et al. [1], and their respective collaborators, could not obtain significative results and, consequently, could not associate the polymorphism to the T2D presence. Chistiakov et al. [22], Mori et al. [26], Pintérová et al. [25], and collaborators had agreeing results with the ones obtained in this meta-analysis, pointing out the polymorphism's protective effect in T2D. Contrariwise Ghoussaini et al. [17] and collaborators sustained that the polymorphism plays a risk factor in the pathology.

Association studies clearly show this gene's regulation in adipose tissue is a complex process. Several studies suggest that genetics or the environment (such as diet) participates in the formation of association patterns of Pro^12^Ala polymorphism with body mass in different human populations. Numerous studies have also clearly showed the heterogeneous effects of Pro^12^Ala polymorphism in decreased risk of T2D several populations. Considering that the two pathologies are multifactorial it is difficult to clarify the real contribution that the polymorphism makes in these metabolic disorders.

The present study includes approximately 11500 individuals, from which 3870 are obese and 7625 are diabetic. From our results we concluded that the Pro^12^Ala polymorphism from PPAR*γ*2 gene has a protective role in T2D but is a risk factor for obesity accordingly with what we should expected substitute to for about to the physiological role of PPAR*γ* itself. It seems that the Pro^12^Ala polymorphism may be a transitory state towards the structural and functional stabilization of the gene role in human kind, resembling a genetic drift at the molecular level.

Therefore, this study supports the need of investment for further research, mainly by population genetics, in order to fully understand the role of this polymorphism in the PPAR*γ* cascades.

## Figures and Tables

**Table 1:** Included studies in the meta-analysis for obesity and respective genotype of the studied population.

Study	Population	Metabolic disorder	Participants	No. of cases (O)	No. of control (NO)	Genotype					
						Pro/Pro		Pro/Ala		Ala/Ala	
						O	NO	O	NO	O	NO
Ghoussaini et al., 2005 [17]	French	Obesity (children)	591	396	195	304	156	84	39	8	0
Ghoussaini et al., 2005 [17]	French	Obesity (adults)	1713	1102	611	857	478	231	123	12	10
González Sánchez et al., 2002 [18]	Spanish	Obesity	459	145	314	119	264	25	50	1	3
Oh et al., 2000 [1]	Korean	Obesity	229	111	118	103	108	8	9	0	1
Ereqat et al., 2009 [19]	Palestinian	Obesity (T2D individuals)	202	121	81	106	73	15	8	—	—
Ben Ali et al., 2009 [20]	Tunisian	Obesity	675	387	288	348	271	39	17	—	—

Summary			**3869**	**2262**	**1607**	1837	1350	402	246	21	14

Legend: T2D: type 2 diabetes mellitus; O: obese; NO: non-obese.

**Table 2:** Included studies in the meta-analysis for T2D and respective genotype of the studied population.

Study	Population	Metabolic disorder	Participants	No. of cases (D)	No. of control (ND)	Genotype					
						Pro/Pro		Pro/Ala		Ala/Ala	
						D	ND	D	ND	D	ND
Ghoussaini et al., 2005 [17]	French	T2D + RIS	946	628	318	511	246	113	63	4	9
Lindi et al., 2002 [21]	Finnish	T2D	490	248	242	163	174	79	61	6	7
Chistiakov et al., 2010 [22]	Russian	T2D + RIS	1185	588	597	401	353	167	208	20	36
Oh et al., 2000 [1]	Korean	T2D	229	58	171	54	157	3	14	1	0
Bouassida et al., 2005 [23]	Tunisian	T2D	488	242	246	216	221	26	23	0	2
Malecki et al., 2003 [24]	Polish	T2D	644	366	278	256	202	99	66	11	10
Pintérová et al., 2004 [25]	Czech	T2D	230	133	97	99	61	31	30	3	6
Mori et al., 2001 [26]	Japanese	T2D	3413	2201	1212	2097	1114	103	96	1	2

Summary			**7625**	**4464**	**3161**	3797	2528	621	561	46	72

Legend: T2D: type 2 diabetes mellitus; D: diabetic; ND: non-diabetic; RIS: reduced insulin sensitivity.

**Table 3:** Sensitivity and specificity for Pro^12^Ala polymorphism and obesity.

Study	TP	FP	FN	TN	Sensitivity	Specificity
Ben Ali et al., 2009 [20]	39	17	348	271	0.10 [0.07, 0.14]	0.94 [0.91, 0.97]
Ereqat et al., 2009 [19]	15	8	106	73	0.12 [0.07, 0.20]	0.90 [0.81, 0.96]
Ghoussaini et al., 2005 [17]	243	133	857	478	0.22 [0.20, 0.25]	0.78 [0.75, 0.81]
Ghoussaini et al., 2005 [17]	92	39	304	156	0.23 [0.19, 0.28]	0.80 [0.74, 0.85]
Oh et al., 2000 [1]	8	10	103	108	0.07 [0.03, 0.14]	0.92 [0.85, 0.96]
González Sánchez et al., 2002 [18]	26	53	314	156	0.08 [0.05, 0.11]	0.75 [0.68, 0.80]

**Table 4:** Sensitivity and specificity for Pro^12^Ala polymorphism and T2D.

Study	TP	FP	FN	TN	Sensitivity	Specificity
Bouassida et al., 2005 [23]	26	25	216	221	0.11 [0.07, 0.15]	0.90 [0.85, 0.93]
Chistiakov et al., 2010 [22]	187	244	401	353	0.32 [0.28, 0.36]	0.59 [0.55, 0.63]
Ghoussaini et al., 2005 [17]	117	68	511	246	0.19 [0.16, 0.22]	0.78 [0.73, 0.83]
Lindi et al., 2002 [21]	85	68	163	174	0.34 [0.28, 0.41]	0.72 [0.66, 0.77]
Malecki et al., 2003 [24]	110	76	256	202	0.30 [0.25, 0.35]	0.73 [0.67, 0.78]
Mori et al., 2001 [26]	104	98	2097	1114	0.05 [0.04, 0.06]	0.92 [0.90, 0.93]
Oh et al., 2000 [1]	4	14	54	157	0.07 [0.02, 0.17]	0.92 [0.87, 0.95]
Pintérová et al., 2004 [25]	34	36	99	61	0.26 [0.18, 0.34]	0.63 [0.52, 0.72]

**Table 5:** Pro^12^Ala polymorphism and obesity.

	Obesity		Total	*P* value
	Obese	Non-obese		
Polymorphism Pro^12^Ala				
Present	423	260	683	*P* < 0.004
Non-present	1837	1350	3187	
Total	**2260**	**1610**	**3870**	

**Table 6:** Pro^12^Ala polymorphism and T2D.

	T2D		Total	*P *value
	Diabetic	Non-diabetic		
Polymorphism Pro^12^Ala				
Present	667	633	1300	*P* < 0.01
Non-present	3797	2528	6325	
Total	**4464**	**3161**	**7625**	

**Table 7:** Estimated risk of the Pro^12^Ala polymorphism with obesity.

	Value	95% CI	
		Low	High
OR for polymorphism Pro^12^Ala (0/1)	1.196	1.009	1.417
OR for variable non-obese = 0	1.113	1.003	1.235
OR for variable obese = 1	0.931	0.871	0.994
Number of valid cases	3870		

**Table 8:** Estimated risk of the Pro^12^Ala polymorphism with T2D.

	Value	95% CI	
		Low	High
OR for polymorphism Pro^12^Ala (0/1)	0.702	0.622	0.791
OR for variable non-diabetic = 0	0.940	0.920	0.961
OR for variable diabetic = 1	1.340	1.214	1.479
Number of valid cases	7625		
